# Severe late onset capillary leak syndrome post allo-HSCT successfully treated by bevacizumab: a case report

**DOI:** 10.3389/fmed.2024.1483064

**Published:** 2025-01-07

**Authors:** Song Xue, Jiaqi Chen, Yuzhu Shi, Lina Zhang, Man Chen, Huipeng Sun, Xingyu Cao, Fang Wu

**Affiliations:** ^1^Department of Bone Marrow Transplant, Beijing Lu Daopei Hospital, Beijing, China; ^2^Department of Laboratory Medicine, Hebei Yanda Lu Daopei Hospital, Langfang, China; ^3^Department of Radiology, Beijing Lu Daopei Hospital, Beijing, China; ^4^Division of Pathology and Laboratory Medicine, Beijing Lu Daopei Hospital, Beijing, China; ^5^Department of Bone Marrow Transplant, Hebei Yanda Lu Daopei Hospital, Langfang, China; ^6^Pharmacy Department, Beijing Lu Daopei Hospital, Beijing, China

**Keywords:** capillary leak syndrome (CLS), allo-HSCT, bevacizumab (avastin), case report, vascular endothelial growth factor (VEGF)

## Abstract

This article describes a case of successful management of late-onset CLS occurring after allo-HSCT, employing bevacizumab as the therapeutic agent. Capillary leak syndrome (CLS) represents a critical complication arising from allogeneic hematopoietic stem cell transplantation (allo-HSCT). The prognosis for CLS remains considerably constrained. A targeted therapeutic approach targeting vascular endothelial growth factor (VEGF) offers a novel strategy for the management of CLS.

## Introduction

Capillary leak syndrome (CLS) is a critical complication of allogeneic hematopoietic stem cell transplantation (allo-HSCT), manifested by weight gain, generalized edema, hypotension, and hypoalbuminemia. The primary pathogenesis of CLS involves damage to the capillary endothelium, leading to a leakage of intravascular fluid into interstitial spaces. Despite the high mortality rate associated with CLS, clinicians continue to face challenges in identifying its triggers, making accurate diagnoses, and devising effective treatments, owing to the incomplete understanding of its underlying mechanisms and the absence of definitive treatment guidelines. Considering the potential of cytokines, particularly vascular endothelial growth factor (VEGF), to cause endothelial injury and increase capillary permeability, VEGF significantly contributes to the pathogenesis of CLS (1). Consequently, the employment of VEGF monoclonal antibodies as a therapeutic modality for CLS presents a promising avenue for effective treatment. Here, we present a case study on the successful management of late onset CLS post allo-HSCT, utilizing bevacizumab as the therapeutic agent.

## Case presentation

In August 2022, a 47-year-old female patient presented to a local hospital with symptoms such as excessive menstrual bleeding, dizziness, and fatigue. Following a comprehensive bone marrow assessment, the patient was diagnosed with AML1::ETO positive acute myeloid leukemia (AML) harboring a KIT mutation. The patient achieved remission following the administration of induction chemotherapy. Nevertheless, in December 2022, during consolidation therapy, the patient experienced disease progression. Remission was re-established after the patient received salvage chemotherapy. On March 9–10, 2023, the patient underwent a matched sibling allo-HSCT subsequent to FLAG/BU/CY preconditioning. After transplantation, there was a consistent reduction in the quantification of the patient's AML1::ETO transcripts level to 0%, accompanied by an absence of significant acute graft-versus-host disease (GVHD) and only mild manifestations of chronic GVHD.

**Figure 1:**
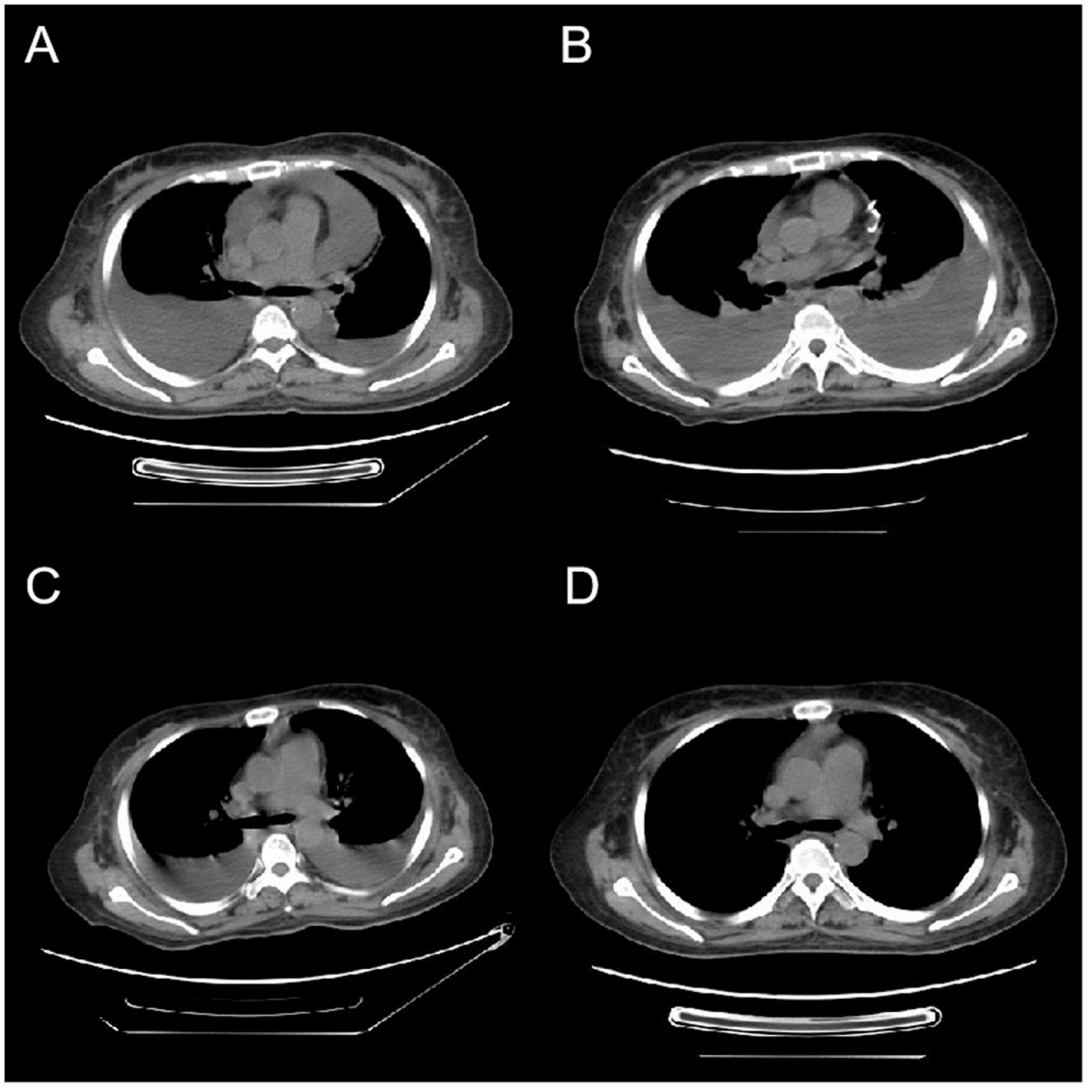
Variations in CT imaging throughout the patient's treatment regimen. **(A)** The initial CT scan upon admission, revealing the presence of bilateral pleural effusions and a pericardial effusion. Four weeks post-admission, **(B)** a CT scan that exhibits an augmentation in pleural effusions from baseline, along with the presence of a visible pericardial drainage catheter. **(C)** A CT scan conducted 3 days following bevacizumab administration, demonstrating a notable decrease in pleural effusions. One week subsequent to the completion of bevacizumab therapy, **(D)** a CT scan indicating the full resolution of pleural effusions.

On January 9, 2024, the patient was re-admitted with oliguria and bilateral lower limb edema. Prior to the onset of novel symptoms, the patient had mild chronic GVHD (skin and mouth involvement, scored as 1 each.) and was managed with low-dose methylprednisolone therapy (0.1 mg/kg). Despite occurring during the COVID-19 pandemic, the patient had not received the COVID-19 vaccine or any other vaccinations. Chest (Figure 1) and abdominal CT scans demonstrated bilateral pleural effusion, ascites, and pericardial effusion. The complete blood count revealed a white blood cell count of 8.90 × 10^9^/L, a hemoglobin level of 114 g/L, and a platelet count of 62.00 × 10^9^/L. The B-type natriuretic peptide (BNP) level was 38.20 pg/ml, plasma albumin quantification was 26.50 g/L and thyroid function tests were normal. Urine analysis revealed mild proteinuria, with a 24-h urine protein quantification of only 499 mg. The patient underwent intravenous administration of methylprednisolone (2 mg/kg), ulinastatin, furosemide/torasemide, and sufficient albumin infusions, but the patient did not respond to these therapy. The patient exhibited pronounced abdominal distension, prompting the execution of abdominocentesis for the purpose of draining excess fluid and alleviating symptoms. The analysis of the ascitic fluid indicated a total cell count of 44 × 10^6^/L, with a white blood cell count specifically measuring 4 × 10^6^/L. The total protein concentration in the ascitic fluid was quantified at 5.8 g/L, while adenosine deaminase (ADA) activity was determined to be 1.80 U/L. Additionally, the quantitative analysis of the AML1::ETO fusion gene of the ascitic fluid yielded a negative result. Although asymptomatic for viral infections, the patient underwent viral testing to identify the cause of the novel symptom. The screening for herpesvirus in ascites samples resulted in negative findings. High-throughput sequencing of ascitic fluid pathogens yielded negative results. The quantitative interleukin-6 (IL-6) level in the ascitic fluid was 529.77 pg/ml. The patient received treatment with hydroxyethyl starch (HES) and tocilizumab, however, no significant therapeutic effect was noted. Given the complexity of this case, multiple rounds of internal discussions were held among specialized experts. Although the patient exhibited significant hypoproteinemia and edema, but did not fulfill the diagnostic criteria for nephrotic syndrome, but the professors participating in the discussions concluded that certain diseases, notably nephrotic syndrome, could not be definitively excluded. Following the patient's inadequate response to glucocorticoid therapy, the professors recommended initiating treatment with rituximab and intravenous immunoglobulin after thorough discussion. The patient, however, remained unresponsive to the treatment. Based on a comprehensive assessment of the patient's clinical symptoms, laboratory results, and imaging findings, a diagnosis of late onset CLS was confirmed in accordance with the established criteria in the literature (3).

**Figure 2:**
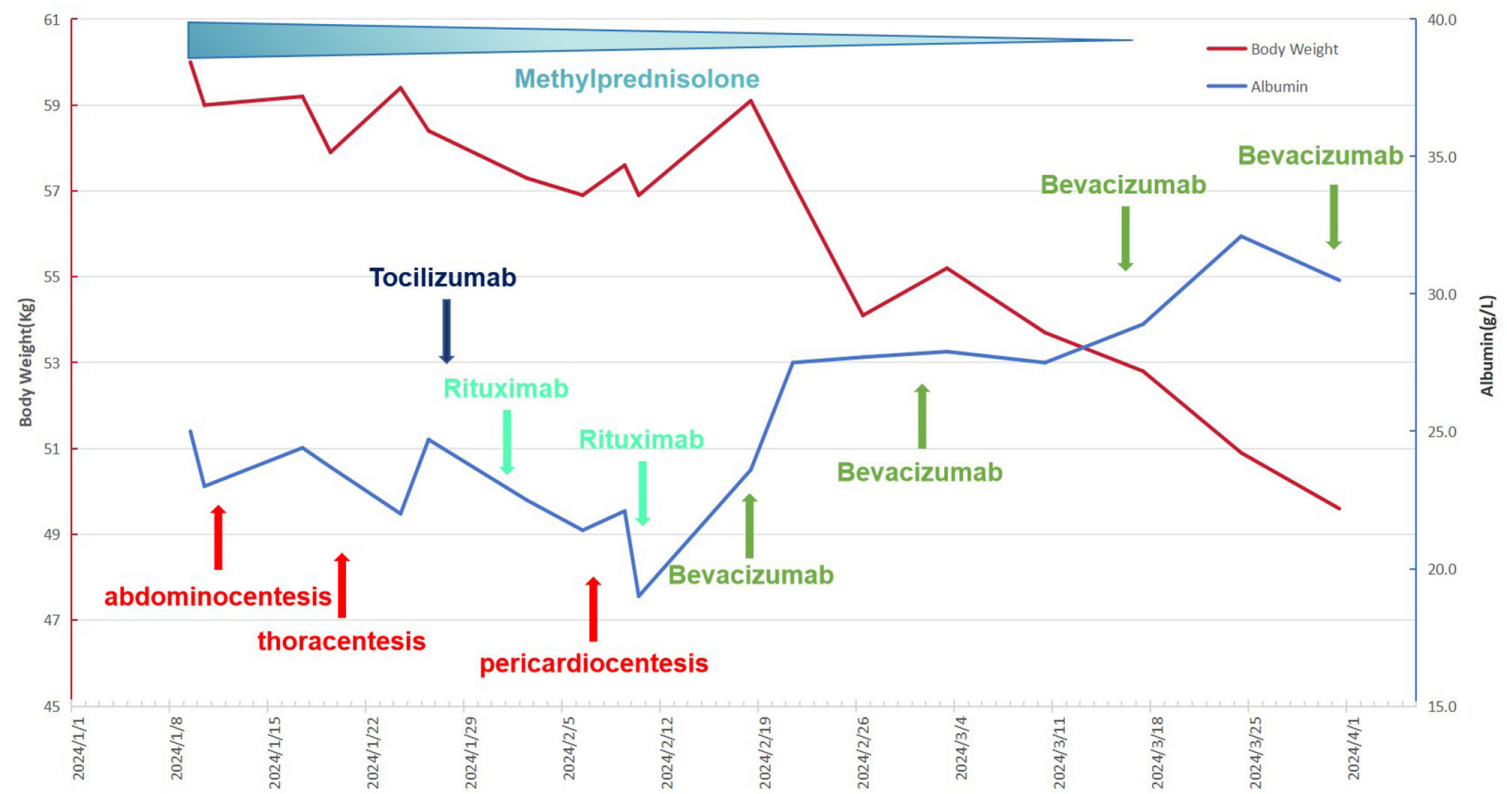
The patient's complete treatment course, including variations in body weight and plasma albumin concentrations throughout the therapeutic process.

The patient exhibited a poor response to all the aforementioned treatments characterized by rapid weight gain, worsening abdominal distension, overt hypotension, tachycardia, and dyspnea. The plasma albumin concentration has diminished to 19 g/L, accompanied by a notable elevation in plasmatic creatinine levels. CT and ultrasound examinations revealed a substantial elevation in pleural, ascitic, and pericardial effusions, the patient underwent thoracentesis and pericardiocentesis procedures aimed at alleviating symptoms related to compression. Considering the patient's critical state and non-responsiveness to other therapies, coupled with the documented success of bevacizumab in treating CLS (2), we initiated a therapeutic regimen involving bevacizumab. The off-label utilization of bevacizumab has been authorized by the Pharmaceutical Affairs Committee of Beijing Lu Daopei Hospital. Separate written informed consent was obtained from the patient before the study, in accordance with the Declaration of Helsinki, and permission to publish the results was granted after the study. This retrospective study was approved by the Ethics Committee of Beijing Lu Daopei Hospital. Bevacizumab (5 mg/kg) was administered intravenously over a 90-min period, to prevent venous thrombosis, the patient underwent subcutaneous injections of enoxaparin. Following treatment, the patient exhibited notable improvement in symptoms, marked by decreased abdominal distension, enhanced urine output, and a substantial reduction in pleural and pericardial effusion drainage. The plasma albumin concentration escalated to 27.5 g/L (Figure 2). A subsequent CT scan, conducted 3 days post-treatment, revealed a pronounced decline in pleural effusion. Subsequently, the patient underwent bevacizumab therapy at a biweekly interval for four cycles, ultimately leading to the complete resolution of pleural effusion. Notably, the patient's pretreatment plasma VEGF level was within normal range (7.9 pg/ml; <142 pg/ml), aligning with previous literature reports (2). During the treatment, the patient's cGVHD in the mouth showed improvement, while stabilization was observed in skin cGVHD. Following the treatment, the patient's clinical symptoms of discomfort resolved, resulting in a high level of satisfaction with the treatment's efficacy.

## Discussion

CLS is a clinical entity defined primarily by weight gain, generalized edema that remains refractory to diuretic treatment, and concurrent hypotension. This condition may result in multiple organ dysfunction secondary to hypoperfusion of blood. CLS associated with transplantation primarily manifests during the early post-transplant period (+10 to +11 days), its emergence may be linked to pre-conditioning toxicity, sepsis, and the use of granulocyte colony-stimulating factor (3). Although the underlying mechanism of CLS remains elusive, hypotheses derived from case reports and case series suggest the involvement of inflammatory factors (4). The literature has documented cases of late onset of CLS, attributable to the variability in triggering factors (5). The present study presents a case report of a patient experiencing a notable delayed onset of CLS 10 months post allo-HSCT. Following a rigorous examination, the emergence of CLS in this patient could not be logically attributed to any factors other than cGVHD. Considering the fundamental nature of cGVHD as a chronic inflammatory disorder, we postulate that the CLS observed in this patient could signify an unusual clinical expression of cGVHD, however, the absence of sufficient direct evidence means that this remains a hypothesis.

The treatment of CLS post allo-HSCT often confronts difficulties in directly targeting the underlying etiology. Consequently, the primary therapeutic strategies emphasize symptomatic management, which aims to alleviate hypotension, diminish inflammation, and sustain perfusion to vital organs. HES is an artificially synthesized colloid solution composed of macromolecules that effectively maintains a stable blood volume over an extended period within the circulatory system. Chinese researchers have documented two successful cases of utilizing HES in the treatment of post-transplantation CLS, thereby suggesting that HES could be a viable option for fluid resuscitation in CLS patients (6). Glucocorticoids and gamma globulin effectively modulate the cytokine storm, mitigate inflammatory responses, and restrict the progression of tissue leakage. Additionally, directly inhibiting the targeted cytokines represents an efficacious approach (7). The patient's plasma IL-6 levels were significantly elevated, prompting the administration of tocilizumab as a therapeutic option. Despite literature reporting significant improvement in CLS with a single dose tocilizumab in rheumatoid arthritis patients (8), our patient exhibited no notable response to this treatment. Drugs used to treat CLS by antagonizing cytokines include colchicine (9) and anakinra (10), however the literature on this subject is extremely limited. Generally, CLS is not a common event, and has a poor prognosis. Despite the availability of numerous potential therapeutic approaches, the prognosis of CLS remains severely limited. A considerable proportion of transplant recipients diagnosed with CLS necessitate admission to intensive care units, and the mortality rate associated with CLS is exceptionally high. CLS significantly influences the overall survival within 100 days post-transplantation and functions as a predictor of transplant-related mortality (3). The patient under investigation displayed the aforementioned characteristics. Despite undergoing various potentially efficacious treatments, the patient demonstrated no therapeutic response, with the condition progressively worsening.

Despite the limited number of relevant case studies (1, 2, 5, 11, 12), targeted therapy against VEGF presents a novel strategy for managing CLS. Bevacizumab has exhibited unanticipated efficacy in select cases of CLS (2, 11); however, a notable proportion of patients remains unresponsive despite elevated plasma VEGF levels (1, 5), in contrast to responders who generally exhibit normal VEGF levels. Despite the confounding clinical manifestations, the grim prognosis associated with CLS necessitates active consideration of bevacizumab therapy upon diagnosis. Bevacizumab treatment, though generally safe, entails an elevated risk of thrombosis, potentially disqualifying its use in managing other transplantation-related vascular complications, including veno-occlusive disease/sinusoidal obstruction syndrome (VOD/SOS) and transplant-associated thrombotic microangiopathy (TA-TMA). In the case we have reported, prophylactic administration of enoxaparin may be appropriate for patients with a low risk of bleeding. For patients intolerant to the adverse effects of bevacizumab, axitinib, a targeted tyrosine kinase inhibitor against VEGF, emerges as a promising therapeutic alternative (12).

## Data Availability

The original contributions presented in the study are included in the article/supplementary material, further inquiries can be directed to the corresponding author.
